# Cell proliferation in the *Drosophila *adult brain revealed by clonal analysis and bromodeoxyuridine labelling

**DOI:** 10.1186/1749-8104-4-9

**Published:** 2009-03-02

**Authors:** Jakob W von Trotha, Boris Egger, Andrea H Brand

**Affiliations:** 1 The Wellcome Trust/Cancer Research UK Gurdon Institute, Department of Physiology, Development and Neuroscience, University of Cambridge, Tennis Court Road, Cambridge, CB2 1QN, UK

## Abstract

**Background:**

The production of new neurons during adulthood and their subsequent integration into a mature central nervous system have been shown to occur in all vertebrate species examined to date. However, the situation in insects is less clear and, in particular, it has been reported that there is no proliferation in the *Drosophila *adult brain.

**Results:**

We report here, using clonal analysis and 5'-bromo-2'-deoxyuridine (BrdU) labelling, that cell proliferation does occur in the *Drosophila *adult brain. The majority of clones cluster on the ventrolateral side of the antennal lobes, as do the BrdU-positive cells. Of the BrdU-labelled cells, 86% express the glial gene *reversed polarity *(*repo*), and 14% are *repo *negative.

**Conclusion:**

We have observed cell proliferation in the *Drosophila *adult brain. The dividing cells may be adult stem cells, generating glial and/or non-glial cell types.

## Background

The generation of new neurons from adult neural stem cells, and their subsequent integration into functional neural circuits in a mature central nervous system, is a widespread phenomenon across the animal kingdom. Adult neural stem cells were initially discovered by Joseph Altman as early as the 1960s [1-3] (for reviews, see [4,5]), and adult neurogenesis has since been shown to occur in all vertebrate species so far examined, including fishes [6-10], amphibians [11,12], reptiles [13,14], birds [15], marsupials [16], non-human primates [17,18], and humans [19].

While adult neural stem cells appear to be common in vertebrates, the situation in insects is much less clear. The house cricket was the first insect in which adult neurogenesis was reported [20], followed by beetles [21,22], cockroaches [23] and moths [24]. However, adult neural stem cells were not found in the locust [21], the monarch butterfly [25], or the honey bee [26]. In vertebrates, adult neurogenesis is thought to be confined to the subventricular zone of the lateral ventricle and the subgranular zone of the dentate gyrus in the hippocampus. In insects, adult neural stem cells were found exclusively in the mushroom bodies (corpora pedunculata; for reviews, see [27,28]), which are implicated in learning and memory, to which adult neurogenesis may contribute [29,30].

In *Drosophila*, adult stem cells have recently been discovered in the adult gut [31,32] and the malphigian tubules [33], but the brain of the adult fly is reportedly devoid of cell proliferation [34]. The neural stem cells (neuroblasts) that generate the central nervous system of adult *Drosophila *are thought to stop division, undergo apoptosis, or differentiate before eclosion [34-37]. Here we show that cell proliferation takes place in the adult *Drosophila *brain. Most of the dividing cells label with Repo, a protein expressed by glia, while a smaller fraction labels with neither Repo nor Elav, a neuronal protein.

## Results and discussion

**Figure 1:**
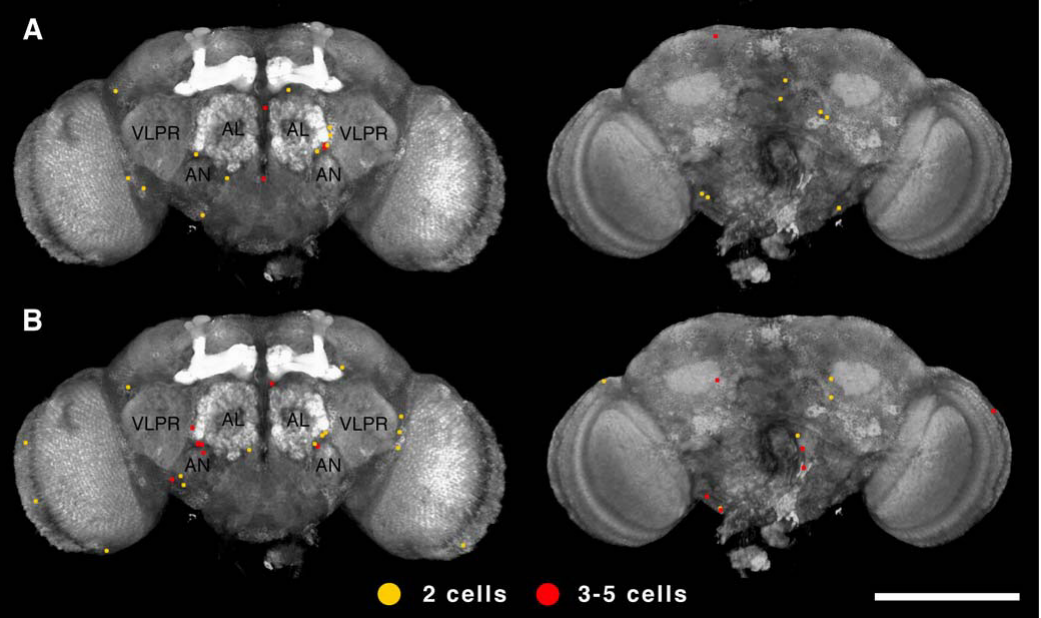
**The distribution of two- and three- to five-cell MARCM clones in control and triple heat-shocked samples**. (A, B) Two-cell MARCM clones (yellow) and three- to five-cell clones (red) from brains of the control (A) (n = 12) and the triple heat-shocked sample (B) (n = 12) mapped onto the common reference brain map. Discs large staining outlines cell cortices and the neuropile. Frontal view, left; caudal view, right. AL, antennal lobe; AN, antennal nerve; VLPR, ventrolateral protocerebrum. The scale bar is 200 μm.

To identify proliferating cells in the *Drosophila *adult brain, we induced MARCM (mosaic analysis with a repressible cell marker) clones [38,39] by expression of hs-FLP recombinase. mCD8-green fluorescent protein (GFP) and H2B-monomeric red fluorescent protein (mRFP) labelled clones were generated in adult flies two days (single heat-shock), or two, four, and six days (triple heat-shock) after eclosion. Female brains were dissected 10 days after eclosion, and clones were mapped onto a common reference brain map (Figure 1).

**Figure 2:**
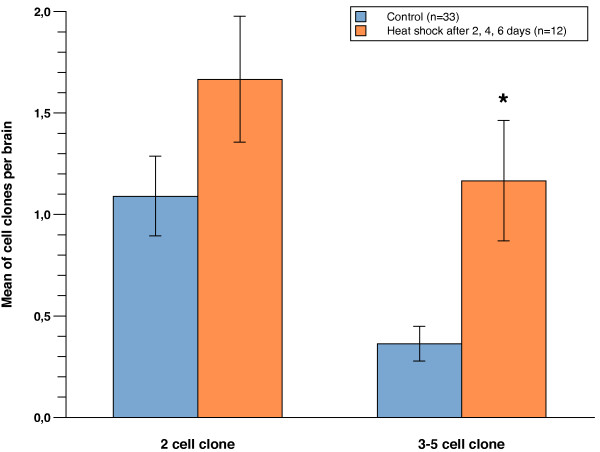
**The frequency of two- and three- to five-cell clones increases after triple heat-shock**. The average number of two- and three- to five-cell cell clones per brain for the non-heat-shocked control (blue; n = 33 brains) and triple heat-shocked animals (orange; n = 12 brains). Error bars denote standard error of the mean; the asterisk indicates *p *< 0.05 (two-tailed) Mann-Whitney *U *and Student's *t*-test.

We observed an increased frequency of clones in the heat-shocked versus the non-heat-shocked control sample, in particular in the number of 2–5 cell clones (Figure 2; Table 1; Additional files 1 and 2). The average number of two-cell clones increased by 50%, from 1.09 ± 0.20 clones per brain in the control (n = 33 brains) to 1.67 ± 0.31 in the triple heat-shocked sample (n = 12 brains). The increase was more pronounced for three- to five-cell clones, where a threefold increase was observed from 0.36 ± 0.09 clones per brain in the control (n = 33 brains) to 1.17 ± 0.30 clones in the triple heat-shocked sample (n = 12 brains). The high background of clones that we observed in the control samples may be due to leaky expression of FLP from the *hsp70 *promoter [40,41].

**Table 1:** The frequency of clones in control and heat-shocked samples

	Control		Heat-shock after 2 days		Heat-shock after 2, 4, 6 days	
	(n = 33 brains)		(n = 12 brains)		(n = 12 brains)	
Clone	Average number of clones/brain	Number of clones counted	Average number of clones/brain	Number of clones counted	Average number of clones/brain	Number of clones counted

1 cell	9.79 ± 0.92	323	12.83 ± 2.02	154	11.92 ± 1.29	143
2 cells	1.09 ± 0.20	36	0.92 ± 0.31	11	1.67 ± 0.31	20
3–5 cells	0.36 ± 0.09	12	0.83 ± 0.24	10	1.17 ± 0.30	14

**Figure 3:**
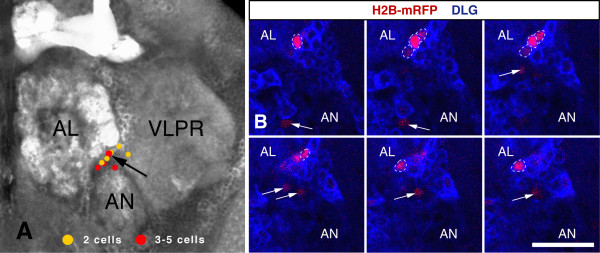
**MARCM clones cluster ventrolateral to the antennal lobes**. (A) Two-cell clones (yellow) and three- to five-cell clones (red) from single heat-shock animals (n = 12 brains) are found ventrolateral to the antennal lobes. The black arrow indicates a four-cell clone that is shown in (B). (B) A z-series of six consecutive, one micron optical sections, running from left to right. The four cells of the clone are marked by H2B-monomeric red fluorescent protein (mRFP) and are outlined with dashed white lines. The white arrows indicate three other single cell clones. Discs large (DLG; blue) outlines cell cortices and the neuropile. AL, antennal lobe; AN, antennal nerve; VLPR, ventrolateral protocerebrum. The scale bar is 20 μm.

A large fraction of the two- and three- to five-cell clones induced by single and triple heat-shock clustered ventrolateral to the antennal lobes. Two-thirds of the three- to five-cell clones on the frontal side of the brain (8 out of 10 clones in the single heat-shock sample and 5 out of 8 clones in the triple heat-shock sample) clustered in this region, and one half of the two-cell clones (6 out of 9 clones in the single heat-shock sample and 5 out of 15 clones in the triple heat-shock sample; Figure 3).

**Figure 4:**
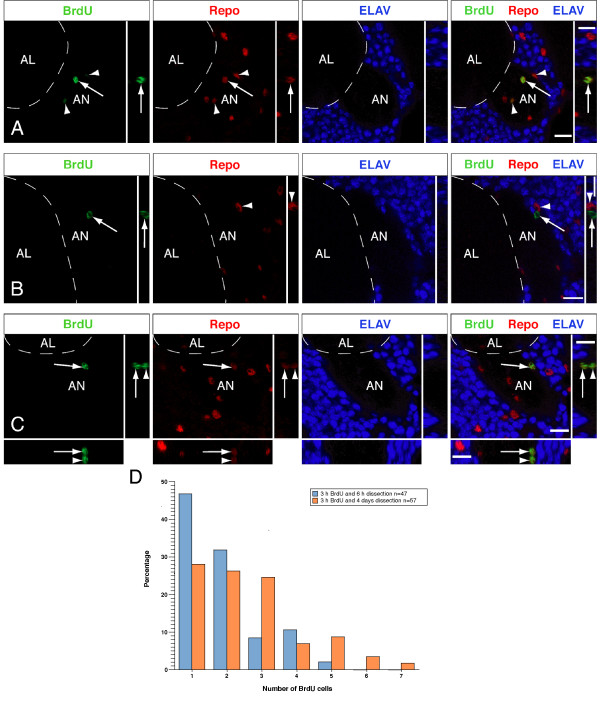
**Most 5'-bromo-2'-deoxyuridine (BrdU)-positive cells co-stain with Repo and localize around the antennal nerve**. (A) Three BrdU-labelled cells in a 3-day-old adult female brain stained for Repo and Elav. One cell can be clearly seen (white arrow) and two others are slightly out of the plane of focus, one more ventro-medial and one more dorso-lateral (white arrowheads). All three cells express Repo but not Elav. Single x-y confocal sections are shown, with insets showing y-z sections; the far right panel shows the merged image. (B) Fourteen percent of BrdU-labelled cells do not express Repo. One such cell (white arrow) is shown here, adjacent to a Repo-positive cell (white arrowhead), in a 10-day-old adult male brain. Single x-y confocal sections are shown, with insets showing y-z sections; the far right panel shows the merged image. (C) Two BrdU- and Repo-positive cells are shown that appear to have divided along the apico-basal axis. The single confocal sections show the more superficially localised cell (white arrow), while x-z and y-z sections (below and to the right, respectively) show the cell lying beneath it (white arrowhead). Neither cell expresses the neuronal marker Elav. Dorsal is to the top in (A, B) and to the upper left in (C); AL, antennal lobe; AN, antennal nerve. The scale bar is 10 μm. (D) A bar graph showing the results of the BrdU pulse chase experiment. Blue bars represent BrdU-labelled cells around the antennal nerve in brains dissected 6 hours after a 3 hour BrdU pulse (n = 47); orange bars represent BrdU-labelled cells in brains dissected after 4 days (n = 57). Note the increase in the number of BrdU-labelled cells in the 4 day sample.

To complement the clonal analysis, we fed 5'-bromo-2'-deoxyuridine (BrdU) to adult flies from 1 day after eclosion onwards and dissected brains 3 or 10 days after eclosion. We detected between one and nine BrdU-positive cells per brain; on average, 3.2 ± 0.3 cells in 3-day-old brains (n = 31 brains) and 4.1 ± 0.5 cells in 10-day-old brains (n = 17 brains). Interestingly, these cells localise around the antennal nerve in an area similar to the majority of two- and three- to five-cell MARCM clones (Figure 4A–C; Additional file 3).

To characterise further the BrdU-positive cells, we stained for Repo (Reversed Polarity, a transcription factor expressed by glial cells) and Elav (embryonic lethal abnormal visual system, a neuronal RNA binding protein; Figure 4). The majority of BrdU-positive cells expressed Repo (86%; 86 out of 98 cells in 3-day-old adult brains, 59 out of 70 cells in 10-day-old adult brains; Figure 4A, C; Table 2). The remaining BrdU-positive cells were Repo-negative and Elav-negative (14%; 12 out of 98 cells in 3-day-old adult brains, 11 out of 70 cells in 10-day-old adult brains; Figure 4B; Table 2).

**Table 2:** The number of BrdU-labelled cells in the *Drosophila *adult brain

	3 days after eclosion			10 days after eclosion		
	(n = 31 brains)			(n = 17 brains)		
	Average number of cells/brain	Number of cells	Percent	Average number of cells/brain	Number of cells	Percent

BrdU (+) Repo (+)	2.77 ± 0.33	86	88 %	3.47 ± 0.48	59	84 %
BrdU (+) Repo (-)	0.39 ± 0.11	12	12 %	0.65 ± 0.14	11	16 %

BrdU incorporation could indicate endoreduplication rather than cell proliferation. To distinguish between these possibilities we performed a pulse-chase experiment (Figure 4D). Adult flies were given a 3 hour pulse of BrdU shortly after eclosion and their brains were dissected after 6 hours, or after 4 days. In the 6 hour sample, only 21% of the brains had greater than one or two labelled cells around the antennal nerve (n = 47 brains). By comparison, 46% of the brains in the 4 day sample had three or more labelled cells (n = 57 brains). Moreover, the presence of six or seven BrdU-positive cells was observed only in the 4 day sample, and not in the 6 hour sample. Of the BrdU-positive cells in the 6 hour sample, 83% were Repo-positive, as were 85% in the 4 day sample.

**Figure 5:**
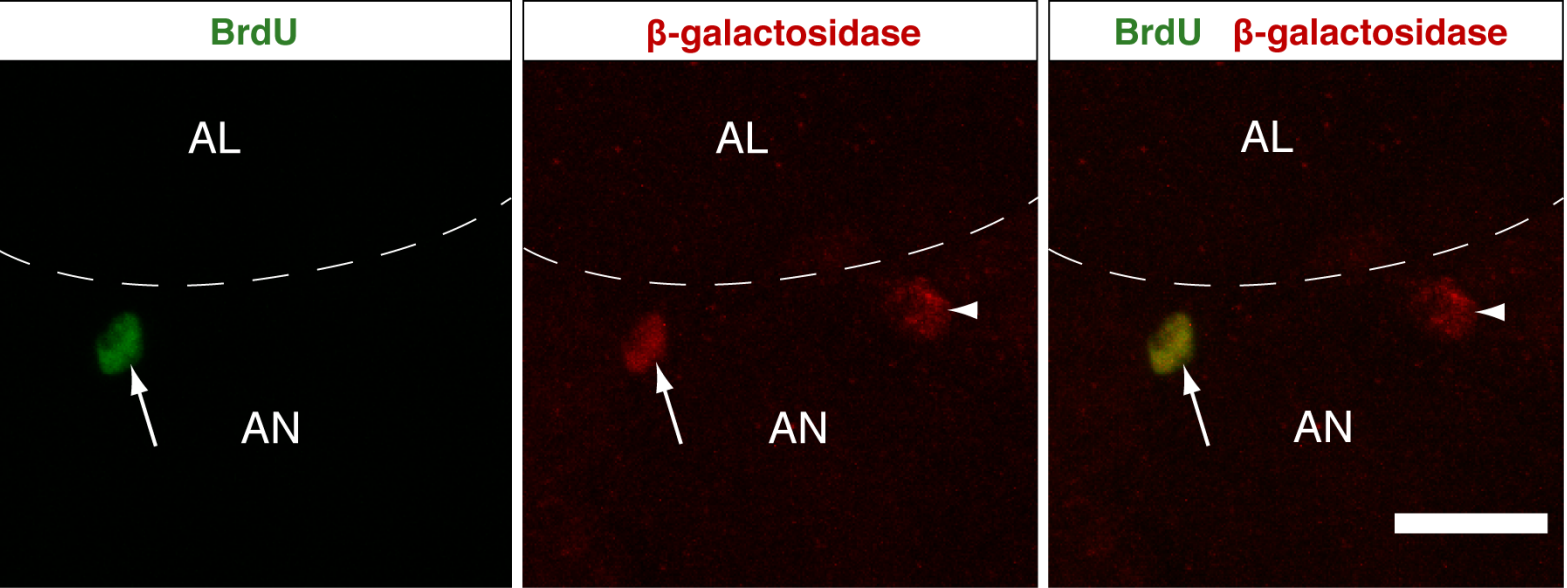
**Mitotic clones around the antennal nerve label with 5'-bromo-2'-deoxyuridine (BrdU)**. Two mitotic, single cell clones are marked by expression of *lacZ *in a 3-day-old adult X15-29/X15-33 brain. One of the two β-galactosidase-positive cells also stains for BrdU (white arrow), while the second does not (white arrowhead). The images are a z-series projection of 24 0.3 μm sections. Dorsal is to the upper left. AL, antennal lobe; AN, antennal nerve. The scale bar is 10 μm.

To detemine whether the BrdU-labelled cells corresponded directly to the dividing cells we observed by MARCM, we combined BrdU labelling with clonal analysis. In order to avoid the high background of clones we observed in the control MARCM samples, we used a different lineage tracing technique that gives few, if any, background clones [31,42]. In 2 out of 18 BrdU-positive brains we observed cell clones (marked by nuclear β-galactosidase) labelled with BrdU around the antennal nerve (Figure 5). We conclude that cells in the adult brain are actively dividing, and that the majority of these cells express Repo.

It is possible that either, or both, of the populations of dividing cells we observed give rise to neurons. Adult neural stem cells in vertebrates, and also in decapod crustaceans, show glial characteristics [43-46]. Astrocyte-like cells in mammals, and radial glia-like cells in non-mammalian vertebrates, act as adult neural stem cells and are responsible for the production of new neurons throughout adulthood. Although we did not observe Elav-positive neurons arising *de novo *in the first 10 days after eclosion, they might appear later in adult life. Alternatively, the BrdU-positive cells might have given rise to Elav-negative neurons.

Most interestingly, the majority of proliferating cells are found in a specific area of the brain, around the antennal nerve. This observation fits with the concept that stem cells are often found in particular microenvironments, or niches [47-49]. In crustaceans, adult neurogenesis occurs in the central olfactory pathway [50-53]. In these arthropods, adult neural stem cells are localised in two discrete clusters on both sides of the olfactory lobe, the equivalent of the insect antennal lobe, and the new neurons produced during adulthood consist of olfactory projection neurons and local interneurons [52,53]. During adult neurogenesis in the subventricular zone of vertebrates, newly produced neurons travel through a rostral migratory stream to the olfactory bulb [4,29]. It has been suggested that blocking adult neurogenesis in crickets impairs olfactory learning and memory [54] and recently, Imayoshi *et al*. [55] showed that, in mice, continued neurogenesis serves to maintain and reorganise the interneuron system of the olfactory bulb.

Until now, adult neurogenesis in insects had been observed only in the mushroom bodies [20,21,23,24]. In *Drosophila*, early [^3^H]thymidine labelling experiments also suggested that cell proliferation takes place in the mushroom bodies of the *Drosophila *adult brain [56], but these results could not be confirmed [34] (this study).

## Conclusion

Here we show that, contrary to earlier reports, there are proliferating cells in the *Drosophila *adult brain. In other insect species adult neurogenesis takes place in the mushroom bodies. We did not observe cell division in these prominent structures, but instead show cell proliferation around the antennal nerve. The majority of proliferating cells expressed Repo (86%), and may be glial, while 14% of the BrdU-positive cells were Repo-negative. A complete lineage analysis and characterisation of the terminal phenotype of the cells generated by these clones will reveal whether glia, neurons, or both are generated in the adult *Drosophila *brain.

## Materials and methods

### Fly stocks and MARCM analysis

*Drosophila *were grown under standard conditions. Oregon R was used as wild-type strain. For the MARCM analysis yw, hsFLP; FRT40A, tub-GAL80/CyO; tub-GAL4/TM6 (a gift of B. Bello) virgin females were crossed to w; FRT40A; UAS-mCD8-GFP, UAS-H2B-mRFP males ([57] and this study). The progeny from this cross were heat-shocked at 37°C for 50 min in a water bath 2 days (single heat-shock sample) or 2, 4, and 6 days after eclosion (triple heat-shock sample). 10 days after eclosion, the brains of both heat-shocked and the non-heat-shocked flies were dissected and prepared for confocal microscopy. Adults of the genotype yw, hsFLP/+; X15-29/X15-33 [42] were used in experiments that combined the clonal analysis and the BrdU labelling.

### Immunohistochemistry and BrdU labelling

Brains were fixed and stained essentially as previously described [58]. BrdU labelling was performed as previously described [34,35]. Adult flies were collected immediately after eclosion and starved for up to 24 hours before feeding on soaked filter paper (Whatman, Springfield Mill, Kent, United Kingdom) and yeast containing 5% sucrose (Sigma-Aldrich, Dorset, United Kingdom), 1 mg/ml BrdU (Sigma-Aldrich, Dorset, United Kingdom) and 1% red food colour (SuperCook, Leeds, United Kingdom). The addition of food colour enabled the selection of flies with sufficient food intake, as monitored by abdominal labelling. For the pulse-chase experiment, adult flies were fed 2 mg/ml BrdU for 3 hours, 15–20 hours after eclosion. The brains from these animals were dissected either after 6 hours or after 4 days. To combine clonal analysis with BrdU-labelling, we used adults of the genotype yw, hsFLP/+; X15-29/X15-33 [42], in which mitotic clones are marked by expression of nuclear β-galactosidase. From shortly after eclosion onwards the flies were fed with 1 mg/ml BrdU. They were subjected to three heat shocks (1 hour at 37°C, followed by a 2 hour recovery phase) on days 1 and 2 after eclosion. The brains were dissected 3 days after eclosion.

Primary antibodies were rabbit anti-β-galactosidase (1:10,000; Cappel, West Chester, Pennsylvania, USA); rat anti-BrdU (1:100; Abcam, Cambridge, United Kingdom); mouse anti-Dlg 4F3 (1:100; Developmental Studies Hybridoma Bank (DSHB), Iowa City, Iowa, USA); mouse anti-ELAV 9F8A9 (1:10; DSHB); rabbit anti-GFP (1:1000; U Mayor and AHB, unpublished); rabbit anti-Repo (1:100; [59]). Secondary antibodies were Alexa488, Alexa568, Alexa633 (1:200; Molecular Probes, Invitrogen, Paisley, United Kingdom).

### Microscopy, cell counting, and image processing

Images were acquired on a Leica TCS SP2 or a Zeiss LSM 510 META confocal microscope. The common reference map for the MARCM analysis is a three-dimensional reconstruction (Volocity, Improvision, Coventry, United Kingdom) of a series of 0.7 μm sections of a 10-day-old adult female brain (20× oil immersion objective, numerical aperture 0.7). Z-series (1.5 μm sections) of brains from heat-shocked and control samples were used for cell counting. Images of control and experimental brains were randomised, and cell counting was carried out double-blind. Images were processed using Volocity (Improvision) and ImageJ [60]. Figures were assembled in Adobe Photoshop 9.0 and Adobe Illustrator 12.0. Statistical analysis was performed using Aabel (Gigawiz, Oklahoma City, Oklahoma, USA).

## Abbreviations

BrdU: 5'-bromo-2'-deoxyuridine; Elav: embryonic lethal abnormal visual system; GFP: green fluorescent protein; MARCM: mosaic analysis with a repressible cell marker; mRFP: monomeric red fluorescent protein; Repo: Reversed Polarity.

## Competing interests

The authors declare that they have no competing interests.

## Authors' contributions

JWT and BE designed and performed the experiments, analyzed the data and wrote the manuscript. AHB conceived the study, designed and supervised the experiments and wrote the manuscript. All authors approved the final manuscript.

## Supplementary Material

Additional file 1 **The frequency of clones with and without heat-shock**. The average number of cell clones per brain for the non-heat-shocked control (blue; n = 33 brains), single heat-shocked (yellow; n = 12 brains) and triple heat-shocked animals (orange; n = 12 brains). Error bars denote standard error of the mean; the asterisk indicates *p *< 0.05 (two-tailed) Mann-Whitney *U *and Student's *t*-test. Click here for file

Additional file 2 **The frequency of clones in control and heat-shocked samples. **The frequency of clones in control and heat-shocked samples Click here for file

Additional file 3 **The location of BrdU-positive cells in the *Drosophila *adult brain.** Shown is a z-series of a 3-day-old adult *Drosophila *male brain. In each hemisphere, four BrdU-positive cells are visible around the antennal nerve (arrows). BrdU, green; Repo, blue; Elav, red. AL, antennal lobe; AN, antennal nerve; VLPR, ventrolateral protocerebrum; SOG, suboesophageal ganglion. Click here for file
