# Supplementary figures and images for: Cell proliferation in the Drosophila adult brain revealed by clonal analysis and bromodeoxyuridine labelling
Source: Neural Dev. 2009 Mar 2;4:9. doi: 10.1186/1749-8104-4-9 (PMC2662830; doi:10.1186/1749-8104-4-9)

Mean of cell clones per brain

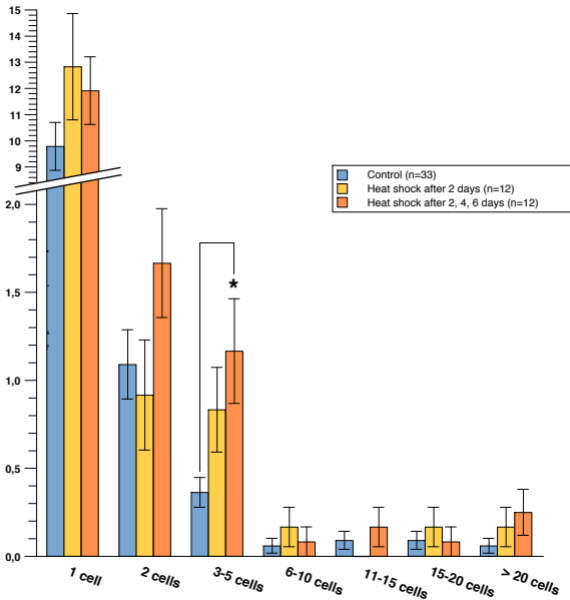

Supplement: Additional file 1 — The frequency of clones with and without heat-shock. The average number of cell clones per brain for the non-heat-shocked control (blue; n = 33 brains), single heat-shocked (yellow; n = 12 brains) and triple heat-shocked animals (orange; n = 12 brains). Error bars denote standard error of the mean; the asterisk indicates p < 0.05 (two-tailed) Mann-Whitney U and Student's t-test. [file 1749-8104-4-9-S1.pdf]
