# Supplementary material for: Cell proliferation in the Drosophila adult brain revealed by clonal analysis and bromodeoxyuridine labelling
Source: Neural Dev. 2009 Mar 2;4:9. doi: 10.1186/1749-8104-4-9 (PMC2662830; doi:10.1186/1749-8104-4-9)
Supplement: Additional file 2 — The frequency of clones in control and heat-shocked samples. The frequency of clones in control and heat-shocked samples [file 1749-8104-4-9-S2.doc]

**Supplementary Table 1**

| **The frequency of clones in control and heat-shocked samples** | | | | | | | | |
| --- | --- | --- | --- | --- | --- | --- | --- | --- |
|  | Control | |  | Heat-shock after 2 days | |  | Heat-shock after 2, 4, 6 days | |
|  | (n = 33 brains) | |  | (n = 12 brains) | |  | (n = 12 brains) | |
| Clone | Average number of clones/brain | Number of clones counted |  | Average number of clones/brain | Number of clones counted |  | Average number of clones/brain | Number of clones counted |
| 1 cell | 9.79 ± 0.92 | 323 |  | 12.83 ± 2.02 | 154 |  | 11.92 ± 1.29 | 143 |
| 2 cells | 1.09 ± 0.20 | 36 |  | 0.92 ± 0.31 | 11 |  | 1.67 ± 0.31 | 20 |
| 3-5 cells | 0.36 ± 0.09 | 12 |  | 0.83 ± 0.24 | 10 |  | 1.17 ± 0.30 | 14 |
| 6-10 cells | 0.06 ± 0.04 | 2 |  | 0.17 ± 0.11 | 2 |  | 0.07 ± 0.08 | 1 |
| 11-15 cells | 0.09 ± 0.05 | 3 |  | 0 ± 0 | 0 |  | 0.14 ± 0.11 | 2 |
| 16-20 cells | 0.09 ± 0.05 | 3 |  | 0.17 ± 0.11 | 2 |  | 0.07 ± 0.08 | 1 |
| > 20 cells | 0.06 ± 0.04 | 2 |  | 0.17 ± 0.11 | 2 |  | 0.21 ± 0.13 | 3 |
